# ASSESSING THE ADEQUACY OF SOCIAL SECURITY RETIREMENT BENEFITS ACROSS RACE-ETHNICITY, GENDER, AND AGE OF RETIREMENT

**DOI:** 10.1093/geroni/igz038.3257

**Published:** 2019-11-08

**Authors:** Jin H Kim

**Affiliations:** Northeastern Illinois University, Chicago, Illinois, United States

## Abstract

This research assessed the adequacy of Social Security retirement benefits across race-ethnicity, gender, and age of retirement, and in turn, whether the differing levels of benefit adequacy have any relation to mortality risk. Prior studies generally find that a replacement rate of between 70 to 80 percent of prior earnings would likely allow a worker to maintain his or her standard of living in retirement since various work-related expenses are reduced or eliminated at the point of transition. As such, the current study used panel data from the 1996 - 2016 waves of the Rand version of the Health and Retirement Study to 1) determine earnings replacement rates for non-Hispanic White, non-Hispanic Black, and Hispanic males and females in the first period of retirement, and 2) to examine whether earnings replacement rates are associated with mortality risk in a Cox regression model. The findings revealed that for those retiring at age 65 or later, Hispanic females and White males had the lowest earnings replacement rates at 39.3% and 40.7%, respectively. For those retiring before age 65, Hispanic males and White males had the lowest earnings replacement rates at 30.3% and 26.7%. Although replacement rates should indeed be lower for high earners due to Social Security’s progressive benefit formula, the low replacement rates determined for Hispanic males and females were unexpected. Moreover, mortality risk was found to be significantly associated with earnings replacement rates in the final model, but the combination of race-ethnicity and gender still showed a stronger relation.

